# Effect of photodynamic therapy and non-thermal plasma on root canal filling: analysis of adhesion and sealer penetration

**DOI:** 10.1590/1678-7757-2016-0498

**Published:** 2017

**Authors:** Marilia MENEZES, Maíra PRADO, Brenda GOMES, Heloisa GUSMAN, Renata SIMÃO

**Affiliations:** 1 Universidade Federal do Rio de Janeiro, Departamento de Engenharia Metalúrgica e de Materiais, Rio de Janeiro, RJ, Brasil.; 2 Universidade Estadual de Campinas, Faculdade de Odontologia de Piracicaba, Departamento de Odontologia Restauradora, Área de Endodontia, Piracicaba, SP, Brasil.; 3 Universidade Federal do Rio de Janeiro, Departamento de Clínica Odontológica, Área de Endodontia, Rio de Janeiro, RJ, Brasil.

**Keywords:** Confocal microscopy, Photochemotherapy, Plasma gases, Root canal filling materials

## Abstract

**Objective:**

The aim of this study was to evaluate the effect of photodynamic therapy (PDT) and non-thermal plasma (NTP) on adhesion and sealer penetration in root canals.

**Material and Methods:**

Sixty single-rooted premolars were used. The teeth were prepared using a crown-down technique. NaOCl and EDTA were used for irrigation and smear layer removal, respectively. The root canals were divided into three groups: control, PDT, and NTP. After treatments, the roots were filled using gutta-percha and either AH Plus (AHP) or MTA Fillapex (MTAF) sealers. Samples were sectioned at 4, 8, and 12 mm from the apex (1-mm slices)and analyzed by the push-out bond strength test (adhesion) and confocal laser scanning microscopy (sealer penetration). Data were statistically evaluated using Kruskal-Wallis, Dunn’s, and Spearman’s tests.

**Results:**

Regarding AHP, bond strength was similar in the NTP group and in the control group, but significantly lower in the PDT group. As to MTAF, both therapies showed lower values than the control group. In the confocal analysis of AHP, maximum and mean penetration, and penetrated area were statistically higher in the control group than in the PDT and NTP groups. Penetrated perimeter was similar among groups. Regarding MTAF, all parameters yielded better results in the NTP than in the control group. The PDT and control groups showed similar results except for penetrated area.

**Conclusion:**

PDT and plasma therapy affected the adhesion and sealer penetration of root canals filled with AH Plus and MTA Fillapex and there is no positive correlation between adhesion and sealer penetration.

## Introduction

The basic requirements for root canal treatment are effective chemomechanical preparation and three-dimensional obturation of the root canal system^1^. The complexity of the root canal system, with isthmuses, ramiﬁcations, and dentinal tubules, makes it impossible to eliminate microorganisms from root canals during preparation^24^. In addition to the routinely used chemical substances and instruments, other technologies have been proposed to promote antimicrobial activity in the root canal system, such as photoactivated disinfection and non-thermal plasma^7,15,20,27,28^.

Root canal obturation is a very important step for a successful treatment. The use of gutta-percha with various root canal sealers is the most common obturation method. AH Plus sealer (Dentsply Maillefer, Ballaigues, Switzerland) is a resin-based sealer widely used for root canal filling due its acceptable physical properties, low solubility and disintegration, apical sealing ability, good adhesion, antimicrobial action, and good biological properties^2^. However, studies have demonstrated AH Plus higher cytotoxic effects compared to MTA-based sealer^30^. MTA Fillapex (Angelus Dental Solutions, Londrina, PR, Brazil) is a calcium silicate-based root canal sealer that contains salicylate resin, diluting resin, natural resin, bismuth oxide, nanoparticulate silica, and MTA. It was developed to utilize the good features of MTA; relatively high levels of biocompatibility, antimicrobial activity, and sealing ability have been reported for this material^1^.

Adhesion and penetration are two important aspects to be considered in sealer selection. Adhesion of an endodontic sealer is deﬁned as its capacity to adhere to root canal walls and promote the union of gutta-percha cones to each other and to the dentin^2,26^. Sealer penetration into dentinal tubules is also a required feature, as it can improve the connection between sealer and dentin^13^. The penetration ability of root canal ﬁlling materials with antibacterial effect into dentinal tubules may also help avoiding colonization by residual bacteria and root canal reinfection^1,6^.

Studies have shown that bond strength and sealer penetration may be affected by the pretreatment of root canal walls and by the type of sealer used^2,11,21^. Regarding the effects of auxiliary technologies used for root canal disinfection, photoactivated disinfection does not adversely affect the bond strength of AH Plus to dentin, but it has a negative effect on MTA Fillapex sealer^17,18^.

This study assessed the effects of photodynamic therapy (PDT) and non-thermal plasma (NTP) on adhesion and sealer penetration in root canals filled with AH Plus and MTA Fillapex and the correlation between adhesion and sealer penetration.

## Material and Methods

### Specimen preparation

Sixty straight single-rooted premolar teeth were used. Teeth with a fully formed apex were selected, whereas roots with resorption defects, fractures, or open apices were excluded. Crowns were sectioned below the cemento-enamel junction so that the lengths of all roots were adjusted to 14 mm using a low-speed diamond saw (Isomet; Buehler Ltd, Lake Bluff, IL, USA) under water cooling. Patency of each root canal was checked using a size 10 K-file (Dentsply Maillefer, Ballaigues, Switzerland) and working length (WL) was established at 1 mm short of the apex. All teeth had their apices sealed with utility wax (Technew, Rio de Janeiro, RJ, Brazil) to prevent flow through them.

Cleaning and shaping were performed with a crown-down technique, using Miltex nickel-titanium rotary instruments (Integra^®^ Miltex^®^, York, PA, USA). The following sequence was used: 35/.10 to prepare the middle-coronal third. The sequence used in the apical third was: 20/.03; 15/.05; 22/.04; 25/.04; 20/.06; and 20/.07. All files reached the WL. Canals were irrigated with 1 mL of 5.25% sodium hypochlorite (Mil Fórmulas, Rio de Janeiro, RJ, Brazil) between each file change. The smear layer was removed after instrumentation with 3 mL of 17% EDTA (Maquira Indústria de Produtos Odontológicos Ltda, Londrina, PR, Brazil), 1 mL *per* minute. Thereafter, the roots were irrigated with 1 mL of distilled water to remove EDTA, followed by 1 mL of sodium hypochlorite. Finally, the root canals were flushed with 5 mL of distilled water and dried with medium paper points (Endo Points, Manacapuru, AM, Brazil). The teeth were divided into three groups (n=20): control (no employment of auxiliary technology used for root canal disinfection), PDT, and NTP.

### Photodynamic therapy

For photodynamic therapy (PDT), after being prepared as described above, the root canals were filled with 15 µg/mL of methylene blue. The solution was then stirred with a sterile #15 K-file (Dentsply Maillefer, Ballaigues, Switzerland) and allowed to stand for 2 min in the root canal (pre-irradiation time). A diode laser (Twin laser, MMOptics, São Carlos, SP, Brazil) was used as a radiation source with total power of 100 mW and wavelength of 660 nm. Optical fiber was initially inserted up to the WL, and spiral movements, from apical to coronal, were performed to allow for adequate distribution of light throughout the root canal. Total irradiation time was 90 s, resulting in an energy of 8 J for each sample, as described by Oliveira, et al.^19^(2015).

### Plasma therapy

A non-thermal atmospheric pressure plasma jet (Plasma Pen™, PVA Tepla America, Corona, CA, USA) and a mixture of helium and oxygen (98% He and 2% O_2_, White Martins, Rio de Janeiro, RJ, Brazil) was used. The gas pressure was kept at 6 bar and 1000 V was applied to generate plasma.

During treatment, the distance between the tip of the plasma jet and the sample was approximately 5 mm. The teeth were exposed to the plasma for 1 min.

### Root canal filling

In control and experimental groups (after photodynamic or plasma therapy), all roots were immediately filled with gutta-percha cones (medium, Microtipped, Endo Points, Manacapuru, AM, Brazil) and AH Plus (Dentsply, Petropolis, RJ, Brazil) or MTA Fillapex (Angelus, Londrina, PR, Brazil) sealers, a total of 6 subgroups (n=10), i.e., control AH Plus, control MTA Fillapex, PDT AH Plus, PDT MTA Fillapex, NTP AH Plus, and NTP MTA Fillapex.

For confocal laser scanning microscopy, each sealer was fluorescently labeled by adding rhodamine B (Sigma-Aldrich, St. Louis, Missouri, USA) at an approximate ratio of 0.1 w/w%^20^. Both sealers were mixed according to the manufacturer’s instructions. A gutta-percha cone covered with sealer was introduced into the root canal. Another medium cone was further used as accessory until the entire length of the root canal was filled. A #45 **McSpadden condenser** (Dentsply Maillefer, Ballaigues, Switzerland) was then used. The plugger was advanced apically up to 4 mm from the apical stop and slowly removed. Afterwards, the plugger was removed slowly whilst being pushed softly against one side of the canal. Roots were sealed with provisional restorative material (Cavitec, Caitech Produtos Odontológicos, Rio do Sul, SC, Brazil). Specimens were kept in an incubator at 37°C and 100% humidity for 2 days.

### Push-out test

Each root was horizontally sectioned with a slow-speed water-cooled diamond saw (Buehler Isomet 2000, Lake Bluff, IL, USA) at 4, 8, and 12 mm from the apex^14^ to produce 1-mm thick slices for each root region (apical, middle, and coronal).

Loading was performed using an electromechanical machine (EMIC DL200MF, São José dos Pinhais, PR, Brazil) at a crosshead speed of 0.5 mm/min until bond failure occurred. Three tips with different diameters were used for load application in the push-out test in the different thirds (0.76 mm for cervical third, 0.60 mm for middle third, and 0.40 mm for apical third). Debonding values (maximum load) were used to calculate the push-out strength in megapascals (MPa), according to the following formula:

Push-out bond strength (MPa) =

$$\mathrm{Push}-\mathrm{out}\;\mathrm{bond}\;\mathrm{strength}\;(\mathrm{MPa})=\frac{\mathrm{Maximum}\;\mathrm{load}\;(N)}{\mathrm{Adhesion}\;\mathrm{area}\;({\mathrm{mm}}^{2})}$$

The adhesion area was calculated by using the following formula: A=π(R + r)[(h^2^+(R-r)^2^]^0.5^

where π=3.14, R is the coronal side radius, r is the apical side radius, and h is the thickness of the slice.

The thickness of each slice was measured using a digital caliper (Vonder, Curitiba, PR, Brazil) and the coronal and apical radii were measured using a stereoscope (Leika MZ75, Meyer Instruments, Houston, TX, USA) and IM50 software (Leika IM50 Image manager, Houston, TX, USA).

### Confocal microscopy

After the push-out test, the remaining gutta-percha was removed and the sections were polished manually with wet 1200-, 2400- and 4000-grit silicon carbide (SiC) abrasive paper (Carbimet Disc Set, Buehler, Lake Bluff, IL, USA). For each abrasive paper, the sections were polished for 1 min.

Specimens were mounted onto glass slides and examined under a confocal laser scanning microscope (Leica Microsystems GmbH, Mannheim, Germany) using a 5x objective (Leica Microsystems GmbH, Mannheim, Germany). Absorption and emission wavelengths for rhodamine-B were set to 540 and 590 nm. Images from each section were taken at a resolution of 1,024x1,024 pixels.

**Figure 1:**
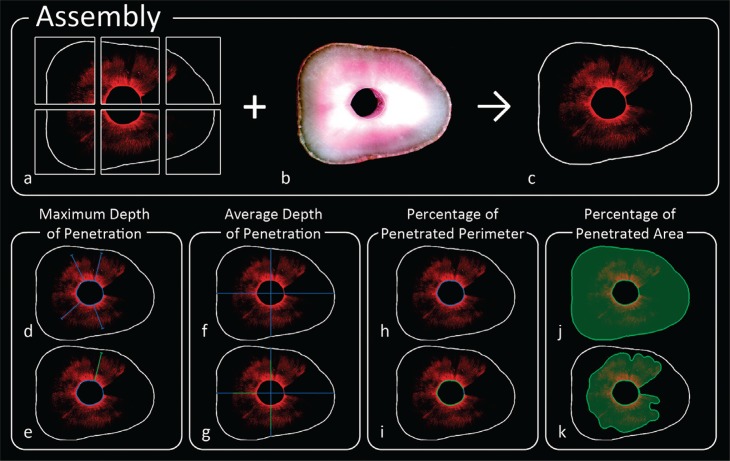
Experimental design: (A) Selection of confocal microscopy images of the chosen slice; (B) Selection of slice image captured with stereoscope; (C) Final image; (D) Maximum penetration into the slice; (E) Selection of the registered maximum penetration (green line); (F) Selection of four points for average penetration depth; (G) Registration of penetration (green line); (H) Measurement of the total perimeter of the canal; (I) Measurement of penetrated perimeter (green line); (J) Total area of the slice; (K) Sealer penetrated area

Image analysis was performed using Adobe Photoshop (Adobe Systems Incorporated, San Jose, CA, USA). Six images were compiled to create an image with the whole tooth. First, confocal microscopy images of the slice were chosen (Figure 1A). Then, the image of the slice captured with the stereoscope was selected (Figure 1B), creating the final image (Figure 1C).

To calculate maximum penetration, measurements of the penetration areas were recorded on the slice (Figure 1D) and maximum penetration was registered (green line) (Figure 1E). For average penetration depth, four points were selected (Figure 1F) and sealer penetration was registered (green line) (Figure 1G). Total perimeter of the root canal (Figure 1H) was measured and the penetrated perimeter registered (green line) (Figure 1I). As to the penetrated area, first the total area of the slice (Figure 1J) was assessed and then the sealer penetrated area was calculated (Figure 1K)^5,8^.

### Statistical analysis

The Kolmogorov-Smirnov test was used to assess the normality of data. Since data were not normally distributed, the Kruskal-Wallis test was used for general comparison and Dunn’s test for pairwise comparison. The Spearman’s test was used to correlate data. The significance level was set at 5%.

## Results

Table 1 shows the median bond strength values (MPa) for both sealers. Plasma therapy results were similar to the control group, whereas PDT presented significantly low strength when AH Plus was used. Conversely, both therapies showed lower bond strength than the control group using MTA Fillapex.

**Table 1:** The median bond strength values (MPa) for both sealers

Section	AH Plus			MTA Fillapex		
	Control (MPa)	PDT (MPa)	Plasma (MPa)	Control (MPa)	PDT (MPa)	Plasma (MPa)
4 mm from the apex	5.67	4.58	4.69	3.55	1.25	2.23
8 mm from the apex	2.99	2.26	2.42	1.66	0.19	0.30
12 mm from the apex	3.32	2.29	3.54	2.18	0.36	0.52
Total	3.33^A^	2.44^B^	3.54^A^	2.22^A^	0.50^B^	0.55^B^

^A, B^ Comparison between groups of the same sealer (Statistical analysis on the row). Different letters indicate statistically significant values

**Figure 2:**
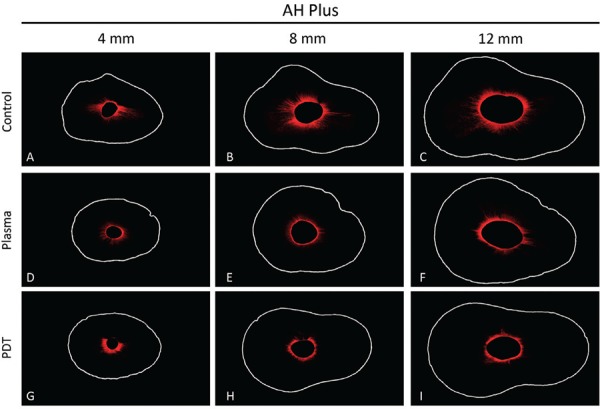
Representative images of confocal for AH Plus sealer

**Figure 3:**
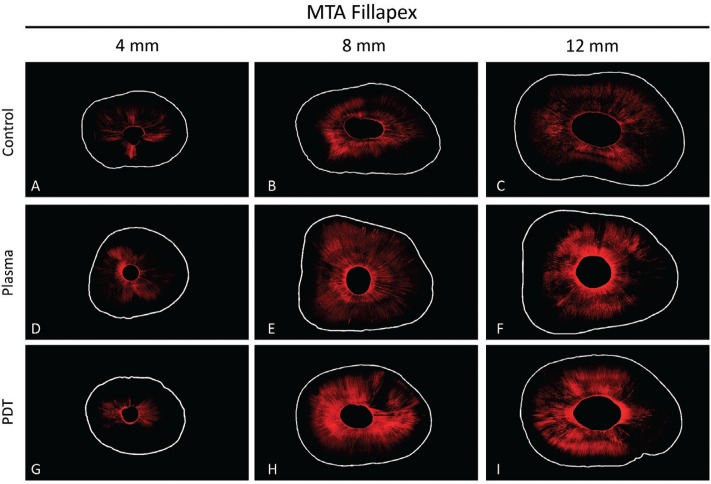
Representative images of confocal for MTA Fillapex sealer

Figure 2 and 3 show representative confocal images of the different groups for AH Plus and MTA Fillapex sealers, respectively.

Table 2 shows median values of maximum and mean sealer penetration depth, penetrated perimeter (%), and penetrated area (%) for the AH Plus sealer. Overall, the control group presented statistically higher values than the PDT and the plasma therapy groups for maximum penetration, mean penetration, and penetrated area, which did not differ among groups. Regarding penetrated perimeter, the groups showed similar values. When the segments were separately evaluated and compared with the overall results, those obtained for the coronal and middle thirds (8 and 12 mm from the apex) were similar to the overall analysis. There was no difference among groups for the apical third. After comparing the different segments, the apical third showed similar or lower values compared to the middle and coronal thirds.

**Table 2:** Confocal analysis for AH Plus sealer

	Group	Maximum depth of penetration (µm)	Mean depth of penetration (µm)	Penetrated perimeter (%)	Penetrated area (%)
Total	Control	1045.95^A^	594.87^A^	82.99^A^	17.76^A^
	PDT	613.04^B^	389.77^B^	98.95^A^	11.18^B^
	Plasma	581.25^B^	398.85^B^	100.00^A^	14.95^B^
4 mm from the apex (apical)	Control	632.71^Ab*^	357.23^Ab*^	61.80^Ab*^	14.10^Ab*^
	PDT	541.90^Aa•^	284.95^Ab•^	58.18^Ab•^	12.02^Aa•^
	Plasma	505.57^Aa♣^	295.17^Aa♣^	83.96^Ab♣^	16.36^Aa♣^
8 mm from the apex (middle)	Control	1192.77^Aa*^	574.44^Aa*^	82.49^Aa*^	19.06^Aa*^
	PDT	684.18^Ba•^	436.32^Aa•^	99.37^Aa•^	11.25^Ba•^
	Plasma	543.41^Ba♣^	417.02^Aa♣^	100.00^Aab♣^	15.28^ABa♣^
12 mm from the apex (coronal)	Control	1154.93^Aa*^	750.78^Aa*^	100.00^Aa*^	25.42^Aa*^
	PDT	641.80^Ba•^	418.91^Ba•^	100.00^Aa•^	9.18^Ba•^
	Plasma	664.50^Ba♣^	448.43^Ba♣^	100.00^Aa♣^	9.90^Ba♣^

(A,B) Comparison between groups; (a,b) comparison between segments in the same group (control*, PDT•, plasma♣)

Regarding MTA Fillapex, plasma therapy showed better results for all parameters than did the control group. PDT group values were similar to the control group for maximum penetration, mean penetration, and penetrated perimeter and similar to the plasma therapy group for penetrated area. The sealer applied to the apical third did not show differences among groups. After comparing the different segments, the values for the apical third were similar or lower when compared to the other groups.

In the push-out test and confocal analysis, the Spearman’s test showed no positive correlation between bond strength and sealer penetration.[Table t3]

**Table 3 t3:** Confocal analysis for MTA Fillapex sealer

	Group	Maximum depth of penetration (µm)	Mean depth of penetration (µm)	Penetrated perimeter (%)	Penetrated area (%)
Total	Control	1,380.47^B^	823.44^B^	82.86^B^	^32.58B^
	PDT	1,398.63^B^	927.50^B^	91.85^A^	40.61^B^
	Plasma	1,645.36^A^	1,290.03^A^	100.00^A^	61.13^A^
4 mm from the apex (apical)	Control	997.51^Aa*^	662.23^Aa*^	75.53^Aa*^	20.73^Aa*^
	PDT	1,147.36^Aa•^	576.71^Aa•^	68.02^Aa•^	18.76^Aa•^
	Plasma	1,268.46^Ab♣^	885.12^Ab♣^	95.54^Aa♣^	42.48^Ab♣^
8 mm from the apex (middle)	Control	1,545.46^Aa*^	937.72^Ba*^	96.31^Aa*^	41.94^Ba*^
	PDT	1,910.25^Aa•^	1,163.26^ABa•^	91.16^Aa•^	49.39^ABa•^
	Plasma	1,884.52^Aa♣^	1,350.20^Aa♣^	100.00^Aa♣^	67.06^Aa♣^
12 mm from the apex (coronal)	Control	1,471.29^Aa*^	868.09^Ba*^	88.47^Ba*^	30.99^Ba*^
	PDT	1,454.64^Aa•^	921.83^Aa•^	99.05^ABa•^	46.29^ABa•^
	Plasma	1,886.04^Aa♣^	1,457.29^Aa♣^	100.00^Aa♣^	63.63^Aa♣^

(A,B) Comparison between groups; (a,b) comparison between segments in the same group (control*, PDT•, plasma♣)

## Discussion

PDT and plasma therapy have been proposed as auxiliary therapy in chemomechanical preparation due to their antimicrobial activity^7,15,20,27,28^. Both technologies create reactive oxygen species, causing serious damage to microorganisms through irreversible oxidation of cell components^12,19,25^. The effects of these therapies have been studied in different periods of time^7,12,15,20,25,27,28^. Although these technologies have shown favorable results concerning their antimicrobial activity, little is known about their impact on adhesion and sealer penetration.

In the present study, PDT was applied for 90 s. This period was chosen because it was the minimum period found in the literature that antimicrobial activity was verified by the same parameters employed in the present study^12,19,25^.

A mixture of helium and oxygen (98% He and 2% O_2_) was applied for 60 s for its antimicrobial properties. Also, this mixture has non-thermal characteristics acting at room temperature and causing no damage to periapical and periodontal tissues. Additionally, short periods are clinically favorable.

Flow and adhesion are essential properties when choosing the proper endodontic sealer. Flow allows adequate penetration of the sealer into the dentinal tubules and may favor contact and confinement of microorganisms to the dentinal tubules, providing better antiseptic action. Adequate flow ability allows filling irregularities, isthmuses, and accessory canals. Adhesion allows the material to remain on the walls, thus aiding stability of the filling mass and preventing microleakage^24^. The present study evaluated the effect of PDT and plasma therapy on adhesion and sealer penetration using two sealers – a resin-based sealer (AH Plus) and an MTA-based sealer (MTA Fillapex). Sealers were also compared for each third and adhesion and sealer penetration were correlated.

With respect to AH Plus, plasma therapy adhesion was similar to the control group, while PDT yielded significantly lower values. An explanation for the poor results of PDT would be the possible interference/remnants of the photosensitizing agent on the dentin surface. On the other hand, plasma therapy was used in dry root canals and had no influence on adhesion. This result contradicts the findings of Ok, et al.^17,18^ (2013,2014), who verified that photoactivated disinfection did not adversely affect bond strength of AH Plus to the root canal dentin. Different results can be associated with different laser systems, photosensitizing agents, and with the segments selected for the push-out test. Regarding the use of plasma, any study had previously evaluated its effect on adhesion, not allowing comparisons with data from the literature.

PDT and plasma showed low bond strength values for MTA Fillapex compared to the control group, showing the negative effect of these therapies on this sealer adhesion. Our results are consistent with those of Ok, et al.^17^ (2013), who verified that photoactivated disinfection adversely affected bond strength of MTA Fillapex. According to these authors, this might have occurred due the type of photosensitizing agent used.

AH Plus showed higher bond strength than MTA Fillapex, in line with Sagsen, et al.^23^ (2011). An explanation for the poor adhesion of MTA Fillapex is that the apatite formed by MTA and phosphate-buffered saline may be deposited within collagen fibrils, promoting controlled mineral nucleation on dentin, seen as the formation of an interfacial layer with tag-like structures^22,23^. Low bond strength of MTA Fillapex could be due to the low adhesion capacity of these tag-like structures^22,23^. Additionally, throughout the experiment MTA Fillapex showed to be quite friable. However, Assmann, et al.^4^ (2012) found similar bond strength comparing AH Plus and MTA Fillapex.

Sealers were manipulated in association with rhodamine. Bitter, et al.^5^ (2009) associated rhodamine with cements and observed that bond strength values were not affected by rhodamine, values were similar to those reported in the literature. The same occurred in the present study, bond strength values found here were similar to those reported in the literature^4,14^.

AH Plus sealing ability was statistically higher in the control group than in the PDT and plasma therapy groups regarding maximum penetration, mean penetration, and penetrated area, and both treatments did not differ between themselves. Regarding penetrated perimeter, all groups showed similar values. However, a different behavior was found for MTA Fillapex. Here, plasma therapy had better results for all parameters than the control group. Maximum penetration, mean penetration, and penetrated perimeter were similar in the PDT and control groups, with similar results for penetrated area in the plasma therapy group. Different results can be related to sealer composition and its interaction with the dentin surface, as well as to different viscosity^16^.

MTA Fillapex penetration was better than that of AH Plus, possibly due to low viscosity and high flow ability of the former^3,16^. These results are in accordance with previous studies^10,16^, however, other studies found similar results when comparing MTA Fillapex and AH Plus^9,24^.

The use of the two sealers in the apical segment did not show differences among groups. The other thirds results were similar to the overall analysis. This difference can be associated with the penetration depth of PDT and plasma. Regarding PDT, the presence of vapor lock may have hindered the action of the photosensitizing agent^29^. In plasma therapy, anatomical limitations due to the distance between plasma pen and apical third may have prevented the action of plasma on the apical third.

The Spearman’s test did not show a positive correlation between adhesion and penetration parameters in any of the sealers studied. Thus, it was verified that while both are important characteristics and the key to root canal filling success^2,6,13,26,30^, good adhesion is not directly correlated with good penetration of AH Plus and MTA Fillapex sealers.

## Conclusion

PDT and plasma therapy affected the adhesion and sealer penetration in root canals filled with AH Plus and MTA Fillapex. Moreover, no positive correlation between adhesion and sealer penetration was found for AH Plus and MTA Fillapex.
